# Health research knowledge translation into policy in Zambia: policy-maker and researcher perspectives

**DOI:** 10.1186/s12961-020-00650-5

**Published:** 2021-03-24

**Authors:** Annie Malama, Joseph Mumba Zulu, Selestine Nzala, Maureen Mupeta Kombe, Adam Silumbwe

**Affiliations:** 1 Department of Health Policy and Management, School of Public Health, University of Zambia, P.O. Box 51110, Lusaka, Zambia; 2 Department of Medical Education, School of Medicine, University of Zambia, P.O. Box 51110, Lusaka, Zambia

**Keywords:** Knowledge translation, Policy-makers, Researchers, Zambia

## Abstract

**Background:**

The translation of public health research evidence into policy is critical to strengthening the capacity of local health systems to respond to major health challenges. However, a limited amount of public health research evidence generated in developing countries is actually translated into policy because of various factors. This study sought to explore the process of health research knowledge translation into policy and to identify factors that facilitate or hinder the process in Zambia.

**Methods:**

This work was an exploratory qualitative study comprising two phases. Firstly, a document review of health policies and strategic frameworks governing research was undertaken to understand the macro-environment for knowledge translation in Zambia. Secondly, key informant interviews were conducted with those responsible for health research and policy formulation. The study interviewed 15 key informants and a thematic analysis approach was used.

**Results:**

The document review showed that there are policy efforts to promote knowledge translation through improvement of the research macro-environment. However, the interviews showed that coordination and linkage of the knowledge creation, translation and policy-making processes remains a challenge owing to lack of research knowledge translation capacity, limited resources and lack of knowledge hubs. Emerging local research leadership and the availability of existing stock of underutilized local health research data provide an opportunity to enhance knowledge translation to feed into policy processes in Zambia.

**Conclusions:**

Public health research knowledge translation into policy remains a challenge in Zambia. To enhance the uptake of research evidence in policy-making, this study suggests the need for improved coordination, financing and capacity-building in knowledge translation processes for both health researchers and policy-makers.

## Background

Globally, it has been acknowledged that health research knowledge translation (KT) into policy and practice is vital for enhancing the performance of health systems. However, evidence indicates that whilst there are numerous promising research findings, they are never translated or take a long time to be translated into health policy [1]. WHO defines KT as “the exchange, synthesis and application of knowledge by relevant stakeholders to accelerate the benefits of global and local innovations in strengthening health systems and improving people’s health” [2]. The focus is on promoting the application of knowledge through a dynamic and iterative process of interactions among the producers and users of research, removing the barriers to research use, and tailoring information to different target audiences to accelerate the societal or economic impact of health research [3].

Across the globe, health professionals face the challenge of translating the best available evidence into actual health interventions and policies in a timely manner to provide the most effective care and services [4]. The gap between existing knowledge and action leads to consequences such as suboptimal use of effective treatments and overuse of ineffective or unproven treatments. This contributes to poor health outcomes, health inequities and a waste of increasingly scarce resources [5]. Consequently, patients fail to gain maximum benefit from advances in healthcare, resulting in a negative impact on quality of life, productivity and resource utilization at an individual and societal level [8]. The field of KT aims to close the research–practice gap through the development of KT strategies which are aimed at aligning healthcare professionals’ decision-making with evidence-based recommendations [6].

The challenge of translating research evidence into policy is evident in most countries. Some of the reasons include policy-makers’ lack of access to research evidence sources, lack of time to review the material, and lack of skills to appraise and understand research evidence [7]. There exists a visible disconnection between policy-makers and public health researchers, compounded by a complex policy environment and the relationships between government officials and academic researchers. Elements of this complexity include diverse understandings of the nature of policy and how research relates to policy, dealing with multiple stakeholders in the policy-making process, and identifying strategies to manage the different cultures of government and academia [8]. Further, the focus with regard to policy-making tends to differ in many aspects between policy-makers and researchers. For example, whilst researchers are concerned with ensuring methodologically rigorous evidence, policy-makers may not focus on this.

Despite the widely documented principle barriers to KT, it is important to note common approaches that enhance evidence uptake which resonate with policy-makers [9]. Researchers ought to adapt the key messages from their research evidence for different policy-makers to facilitate adoption [5]. Other studies encourage the building of partnerships between researchers and policy-makers that allow for dialogue and trust-building around the tasks of asking and answering relevant research questions [10]. Further, bridging the gap between health policy-makers and researchers may require involving both parties in planning and execution of health research, institutionalizing research and ensuring that researchers pay attention to the needs of policy-makers [11]. Even with some of these recommendations, engagement between health policy-makers and researchers remains slow, particularly in low- and middle-income countries, which affects the rate at which research evidence influences policies to improve population health.

In Zambia, the field of KT has evolved over the years, particularly in the health sector, where the Ministry of Health (MoH) has created a specific position for a KT expert. Policies and programmes have been initiated to provide guidance for national-level KT efforts [12]. This includes the establishment of the National Health Research Authority (NHRA), a regulatory body which was created under the National Health Research Act, 2013 [No. 2 of 2013] to promote, regulate and coordinate ethical conduct of quality health research and facilitate evidence-based policies and programmes that improve population health [13]. Despite the stated progress, studies on KT in Zambia have remained scarce, particularly with regard to understanding the context within which the process occurs. This study sought to explore the process of health research KT into policy and to identify factors that facilitate or hinder the process in Zambia.

## Methods

### Study design

A qualitative case study design comprising a document review and key informant interviews was adopted. The case study design helped to describe the KT process, the context in which it occurs and to identify related factors [14]. The knowledge-to-action (KTA) framework was used to explore the process of KT [15]. National-level policy documents were reviewed to assess the degree to which the policy environment is enabling health research KT in Zambia.

### Study setting and participants

The study was conducted in Lusaka, the capital city of Zambia, because this is where most national policy-makers and public health researchers are based. Study participants comprised those who had worked for at least 1 year in their respective organizations and were responsible for conducting health research and/or were involved in the process of health policy formulation. They were required to at least have had experience in conducting studies that in one way or another informed government health policy.

The key informants included policy-makers and representatives from various public health research organizations within Zambia, with headquarters in Lusaka District, that were involved in generating evidence, regulating health research and implementing health programmes (Tables 1 and 2). Among the organizations included were the NHRA, the University of Zambia School of Public Health (UNZASoPH), Zambia National Public Health Institute (ZNPHI), Southern Africa HIV and AIDS Information Dissemination Service (SAfAIDS), Centre for Infectious Disease Research in Zambia (CIDRZ) and National AIDS Council (NAC), MoH and the National Assembly Parliamentary Health Committee.

**Table 1:** Key informants: policy-makers/regulators/implementers

Participants	Organization
Key informant #3	Parliament
Key informant #7	MoH
Key informant #8	MoH
Key informant #9	MoH
Key informant #11	NHRA
Key informant #13	Parliament
Key informant #14	MoH
Key informant #15	Parliament

*MoH* Ministry of Health; *NHRA* National Health Research Authority

**Table 2:** Key informants: knowledge creators

Participants	Organization
Key informant #1	CIDRZ
Key informant #2	UNZASoPH
Key informant #4	NAC
Key informant #5	ZNPHI
Key informant #6	SAfAIDS
Key informant #10	NHRA
Key informant #12	NHRA

*CIDRZ* Centre for Infectious Disease Research in Zambia; *UNZASoPH* University of Zambia School of Public Health; *NAC* National AIDS Council; *ZNPHI* Zambia National Public Health Institute; *SAfAIDS* Southern Africa HIV and AIDS Information Dissemination Service; *NHRA* National Health Research Authority

### Sampling methods

The study utilized both purposive and snowball sampling techniques in selecting participants in order to achieve a maximum-diversity sample across the targeted population. We had anticipated that 20 key informants were enough to gain the information sought. However, a total of 15 key informants were interviewed because information saturation was reached. The term saturation commonly indicates that, based on the data collected or data analysed thus far, further data collection and/or analysis is unnecessary [16].

### Data collection method

We first reviewed key health policy documents to become familiar with how the policy and legal environment in Zambia affects the translation of evidence into policy. The health policy documents and strategies were systematically selected from MoH, as the ministry governs health research and sets the direction for evidence-based decision-making in the country. Key informant interviews were then conducted, which were aimed at collecting data from participants who were experts as well as key stakeholders in health research KT. Tables 1 and 2 show the key informants interviewed. The interviews varied according to whether the participant was a policy-maker or health researcher in their respective organization. The key informants were individually contacted and asked to participate in the study. When they agreed, an appointment was set. Both face-to-face and phone interviews were employed. A voice recorder was used to capture the data, with consent from the participants. After each interview, the key informants were asked to suggest names of people who would provide more information on the research topic. The researchers then contacted the suggested names and proceeded to schedule an interview. The data were collected over a period of 3 months.

### Data analysis

A thematic analysis approach, which is the process of identifying themes and patterns within the data, was utilized using NVivo 12 software [17]. Initially, the transcripts were read to become familiar with the data, at the same time paying attention to emergent themes addressing KT and factors shaping the process. Useful notes from the field reports were extracted and themes generated. A predetermined coding structure consisting of the adapted core thematic areas of the KTA framework was used to explore the KT process [15]. Once all data were coded in their respective themes, code reports were then generated and analysed.

### Ethical considerations

This study received ethical approval from the University of Zambia Biomedical Research Ethics Committee (UNZABREC) as well as the NHRA. Relevant approvals were also obtained from the MoH. All the participants in this study provided consent. Information sheets detailing the study description were shared with all participants prior to the interviews, and signed consent was obtained.

## Results

The qualitative results are presented in two parts: firstly, the document review that details the positions of various national health research governing policy documents on KT (Table 3); and secondly, the key informant interview data, which are presented according to policy-maker and researcher perspectives with respective verbatim quotes. The data detailing the KT process are organized according to key constructs of the adapted KTA framework to explain the KT process [15]. The KTA framework has two cycles: firstly, the knowledge creation cycle, which consists of actors and roles in KT, knowledge synthesis, knowledge products, dissemination and priority-setting; and secondly, the knowledge application cycles, which consist of adoption and adaptation of research findings as well as monitoring and evaluation. The factors shaping the KT are categorized as barriers or enablers (Table 4).

**Table 3:** Health research governing policy documents in Zambia

Title	Year	Position on use of evidence for policy
The Zambia National Health Research Policy	2010	Set specific policy measures on research priority-setting. Seeks to provide mechanisms and criteria for identifying and agreeing on national health research priorities
Zambia National Health Policy	2012	Emphasized the need to improve dissemination and utilization of research findings, the need to increase funding levels for local research and prioritize health research amid scarce resources
Zambia National Health Research Act, 2013 [No. 2 of 2013]	2013	Provides power by law to the National Health Research Authority to regulate and coordinate all health research in Zambia and promotes the translation of health research into policy
National Health Research Authority Strategic Plan	2018–2021	Seeks to promote health research through effective regulation coordination, capacity-building and knowledge translation
Zambia National Health Research Agenda	2018–2021	Aligns the production of research evidence to the national health goals and objectives and also provides guidance to researchers, research institutions, policy-makers, programme implementers and other partners

**Table 4:** Key thematic categories

Main theme	Sub-theme
Knowledge creation	Actors and roles in knowledge translation Knowledge synthesis Knowledge products Dissemination of findings Research priority-setting
Knowledge application	Adoption and adaptation of research findings Monitoring and evaluation of research evidence
Facilitators of knowledge translation	Emerging leadership to support local research Availability of locally collected data
Barriers to knowledge translation	Limited knowledge translation capacity Limited resources for research Lack national knowledge management hubs

The reviewed policy documents seek to promote translation of health research into policy by providing a legal framework to regulate and coordinate all health research activities in Zambia, alignment of research evidence to the national health goals and objectives, and development of mechanisms and criteria for identifying and agreeing on national health research priorities. However, these policies seem to focus more on improving the research environment, with rather unclear guidelines on how to ensure that health research knowledge is translated and adopted in national health policies by policy-makers. The policy documents only refer to limited aspects of KT. Further, the policies do not state what roles should be played by various actors in the KT process to ensure that evidence is incorporated in national health policies.

### Research knowledge creation

#### Actors and roles in health research KT into policy

The major actors identified as being directly involved in the process of translating research evidence into policy are the researchers who generate the knowledge, the government through the Executive, Legislature and the Judiciary as well as the policy-makers from the MoH, and other stakeholders such as the media, funders and the communities who advocate for policies to be formulated based on available evidence. Some respondents, however, indicated that there are different roles that each actor plays depending on the stage of KT and the policy process. Relationships among the key actors in the KT and policy process play an important role in influencing the embracing of evidence in national public health policies. One policy-maker stated:

*The main actors are the researchers themselves, the health workers who are the implementers and policy-makers, but there are many stakeholders, as well as the Ministry of Health who is ourselves. This is because what is likely to happen is that, if a researcher conducts their research, the next thing they want to do is to inform Ministry of Health of the results. This is important; they have to engage the Ministry of Health because we are the ones who proceed to do any implementation.* [KI #8, Policy-maker]

Another policy-maker added:

*It depends with the kind of policy to be formulated and at what stage of the policy process. If it’s change of policy on, say, user fees for health services and introduction of health insurance, the ministerial cabinet and other arms of government will play an important role in the policy formulation at that level.* [KI #14, Policy-maker]

#### Knowledge synthesis

Some researchers indicated that most research institutions had limited capacity to synthesize health research findings because they were inadequately trained in synthesis techniques. A major impediment to conducting research synthesis was the inherent time, effort and resources required to undertake such an activity. Most research conducted was merely for academic purposes, with limited emphasis on methodological rigor, which compromised the quality of evidence. In addition, a large proportion of research was donor-funded, and when research is externally funded it is sometimes difficult to access the findings owing to data restrictions in the contractual agreements. This meant that during synthesis, researchers may not have access to certain kinds of evidence, and even when they do, the quality may be an issue. It was the view of the researchers that most local health research institutions are not set up to conduct data synthesis, as one researcher noted:

*Knowledge synthesis is very important, and part of the reason why knowledge translation in this country is poor is because research institutions are not set to synthesize knowledge. We have a lot of data that are collected in vain and are left to waste. We have students, academicians from outside Zambia accessing that data, translating and publishing it into papers, while the Zambians are simple data collectors.* [KI #1, Health Researcher]

#### Knowledge products–research outputs

Health researchers agreed that they produced knowledge in various research programmes that included sexual reproductive health, HIV, tuberculosis (TB) and malaria, among others. Some research organizations had particular strength in conducting clinical trials and evaluating HIV vaccines, and worked on endemic diseases such as shigella, cholera, typhoid and *E. coli*. Much health research knowledge is generated locally, some of which has been used to inform policy, as some health researchers noted regarding their focus areas of knowledge creation:

*Our research centres around communicable diseases (HIV, hepatitis, TB) and noncommunicable diseases (diabetes, hypertension).* [KI #9, Health Researcher/Policy-maker]

Another health researcher had this to say:

*We do various health research programmes, clinical trials, we evaluate HIV vaccines and drugs, and now we are working on HIV virus vaccine, as well as TB.* [KI #1, Health Researcher]

The most frequently mentioned element of health research KT into policy was the development of policy briefs that are shared and debated with the relevant technical working groups (TWGs). These TWGs consist of experts in the key fields, who interrogate key recommendations from the policy briefs on such aspects as operational feasibility and relevance to the local context. The TWGs are then responsible for engaging and guiding the MoH to take relevant policy action. Some policy-makers noted that once the policy brief has been discussed, a policy dialogue then ensues. The dialogue involves a large number of stakeholders and continues for some time until a decision is made about whether some recommendations can be incorporated into policy. However, this process unfolds differently depending on the context, and in some instances may not happen for certain health policies, as one policy-maker/researcher explained:

*Once the research has been done, there is a development of what we call a policy or technical brief. So once a policy brief has been developed, then that is basically submitted to the Ministry of Health usually through the technical group. Once that stage of submitting the policy brief to the technical group has passed, then it goes to the senior management of the Ministry of Health; then the senior management will discuss that issue, and then if they find that there is sufficient evidence they will proceed with guiding the policy formulation.* [KI 11, Policy-maker/Health Researcher]

#### Dissemination of research findings

Some health researchers indicated that they did not make their research findings available to policy-makers because they were not obliged to do so and policy-makers did not ask for the evidence. However, they used various avenues including conferences, meetings and journals for dissemination of the health research findings. It was reported that even when researchers did disseminate information, it was difficult for policy-makers to use some of the research findings because of the way it was communicated. Sometimes the researchers did not communicate their findings clearly, as their reports were filled with technical language, making it difficult for interpretation and use by policy-makers. The policy-makers highlighted the need for research information to be presented in actionable format. Further, it was suggested that researchers ought to prioritize dissemination of findings locally as opposed to international platforms. One of the researchers remarked:

*What has been happening is that a lot of people conducting research are academicians, and so a lot of times they opt to publish their results in high-impact factor journals so that the paper is seen by the world. For sure we are not disseminating that information adequately within our local platforms so as to influence the different policy agendas that happening.* [KI #12, Health Researcher]

A policy-maker also recounted:

*With knowledge translation, the method of communication is really key, so we would disseminate, but most of the time when we do the study, we have the conclusions. You may have the recommendations but the recommendations may not be as useful, because sometimes the researchers are not describing how these recommendations can be operationalized, which makes it difficult for the policy-makers to consider this evidence.* [KI #15, Policy-maker]

The policy-makers and health researchers acknowledged that dissemination is an important step in KT to inform evidence-based policies. However, for the research results to be utilized, it is important that they are strategically disseminated to those that make policy decisions. It was reported that there is a directorate of policy and planning within the MoH that seeks to ensure that health policies are evidence-informed. Such departments should not be left out when disseminating research findings that may have wider health policy implications, as a policy-maker/researcher explained:

*Within the Ministry of Health, we have directorates of policy and planning, which are very influential in shaping ministerial policy decisions. Such a department should be strategically engaged during dissemination if our research findings have to be adopted in public health policies. We should not only disseminate, but as well follow up with the policy-makers to ensure a better understanding of what we are proposing as researchers.* [KI #8, Policy-maker/Health Researcher]

#### Research priority-setting

The key informants revealed that there were no clear research priority-setting mechanisms in most institutions. Both the researchers and policy-makers felt that external funding dictates the local research agenda and/or priority-setting in their respective institutions, which ultimately affects how policy-makers receive or perceive the evidence provided to them. Policy-makers may sometimes perceive externally funded research findings as not promoting national interests, and hence disregard them, however important. Whilst funders may have greater influence on local research priorities, it was also mentioned that some studies are commissioned based on programme implementation gaps as well as their public health relevance. A health researcher explained:

*We don’t have indigenous funding of our own, so we cannot determine that we are going to research on this. Our researchers respond to competitive calls for funding, and those calls come with already predefined areas. So I think while we might be talking about setting priorities in research, that is an effort in futility. You only set priorities when you have money to fund those priorities; we respond to funds.* [KI #1, Health Researcher]

One policy-maker remarked:

*Most research that is conducted is sponsored by donors; therefore, only research in which donors have interest is sponsored and undertaken, thereby leaving the other research that the government would want to research on.* [KI #13, Policy-maker]

Another policy-maker who is also a health researcher indicated:

*It is very difficult to come up with the priority areas because there is a degree of inter-relation. However, you all know that we have got partnerships that help us to combat some of these communicable diseases such as HIV, so that makes it easy for priority-setting because some of the top ten communicable diseases are of public health significance.* [KI #9, Health Researcher/Policy-maker]

### Research knowledge application

#### Adoption and adaptation of research findings to local context

Some policy-makers and researchers shared experiences of when research findings were used to inform policy. One such example was the integrated management of malaria and pneumonia study that was conducted in Chikankata, Southern Province of Zambia, in 2009. The study was looking at community health workers’ capacity to diagnose and treat both malaria and pneumonia. The results of the study were used to inform the policy on integrated community case management of malaria, pneumonia and diarrhoea. However, both the policy-makers and researchers mentioned that even when evidence is adopted, there are not adequate monitoring mechanisms for monitoring the performance of the evidence-informed policies.

*In our study, the first thing we did was the presentation of findings to the child health technical working group. After discussing the findings and the recommended policy options, it was found to be of value. The Ministry of Health agreed to implement what is now called the integrated community case management of malaria, pneumonia and diarrhoea. The decision was made that community health workers can prescribe amoxicillin and utilize the rapid diagnostic test to diagnose malaria. We advocated for this policy because there had been overwhelming evidence this could be done by the community.* [K4 #13, Health Researcher]

### Facilitators of research KT

#### Emerging leadership to support local research

Both the health researchers and policy-makers were of the view that there is political will from the leadership at the MoH to accord the required importance to research KT. To this effect, the MoH has facilitated the creation of the NHRA to coordinate research and facilitate KT. The MoH has undergone various reforms over the years in order to reposition itself for better management and use of research evidence and translate it into policy by providing a legal framework. This has culminated in the creation of the KT officer’s position within the MoH. However, it was noted that this position still required more technical and financial support to work more effectively. This should include the capacity to manage all forms of knowledge, including programmatic knowledge, across the various departments of the MoH to inform policy.

*One of the ingredients that are leading knowledge translation process is mainly leadership and the environment, because we have had research since time in memorial. Because research is a big thing, it needs to stand on its own. Some of the factors enhancing knowledge translation is mainly related to what is being envisioned by leadership, which I think is what should be the case.* [KI #8, Health Researcher/Policy-maker]

#### Availability of locally collected data

Locally generated knowledge was reported to have a greater impact on policy-making because it was tailored to the local needs. Some health researchers noted that the volume of locally generated research had been increasing, which meant that the evidence base is widening. There exists a large pool of routinely collected data in the health information management systems that is underutilized, yet may provide critical evidence to inform policy. However, it was stated that local research was too fragmented, in that researchers doing similar work sometimes present conflicting results, which negatively affects evidence use in policy-making, as one of the health researchers remarked:

*We could use more of the already existing locally collected data. We do have a lot of data sources but they are so fragmented. The Ministry of Education has information system, Ministry of Health, National AIDS Council, etc. Also, every researcher is doing their own things with some otherwise conflicting findings on key topics. This defragmentation makes it hard for the policy-makers.* [KI #4, Health Researcher/Policy Advocate]

### Barriers to research KT

#### Limited KT capacity

Most researchers and policy-makers were of the view that there was limited capacity and understanding of the various facets of KT among both the researchers and policy-makers. Enhancing skills needed for conducting research and KT were identified as key to improving the uptake of evidence in Zambian health policies. Continuous capacity-building on the different elements of KT among knowledge translators and policy-makers, as well as development of effective communication strategies, should be prioritized. It was indicated that building the capacity to generate knowledge that is useful at different stages of the policy process is needed. In addition, it was stated that capacity-building for policy-makers will enable them to engage effectively with the health research findings, as one health researcher/policy-maker noted:

*It is very important to have a skill as you embark on certain processes that are both research itself and knowledge translation. So, in terms of skills, I mentioned that a lot of people have gotten education, but we are still a growing country so we may still have gaps here and there in terms of skills to conduct research, but also at the level of policy translation its either insufficient or inadequate.* [KI #7, Health Researcher/Policy-maker]

Another policy-maker/health researcher stated:

*[The] capacity to generate the knowledge, analyse the data in such a way that it is useful for policy-makers, synthesize that research knowledge into a form that can be used into policy formulation, is the key, as well as the capacity of the policy-makers themselves to appreciate the research knowledge and be able to utilize that knowledge for policy formulation.* [KI #11, Policy-maker/Health Researcher]

#### Limited resources

Health researchers and policy-makers stated that lack of resources is an impediment to the process of translating health research knowledge into policy. Researchers stated that research is expensive, and thus having the resources to conduct research is one of the greatest challenges. Resources refer not only to financial support, but also equipment and infrastructure. The lack of resources constrains the research process including the outputs, which limits the chances of the findings being translated into policy. Furthermore, it was suggested that government should increase funding to research. One health researcher indicated:

*Research is not a cheap exercise and for my capacity to be felt as the researcher, it requires some resources, qualified human resource, it will require infrastructure if you have to do more especially in the health sector, the health sector is really different from maybe commerce. In the health sector if you have to do meaningful research, it has to be pure science, so it is expensive for all those equipment and chemicals.* [KI #10, Health Researcher]

A policy-maker had this to say concerning the limited resources in carrying out health research:

*Research knowledge is sometimes inaccurate due to limited funds to finance health research.* [KI #13, Policy-maker]

#### Lack of a national knowledge hub for health research findings

The participants indicated that Zambia lacked a national knowledge hub for all health research findings generated locally. Some health researchers and policy-makers reported that there is research conducted in many parts of the country, but only research from key provinces such as Lusaka and Copperbelt may come to the attention of policy-makers, while other research findings from other districts, however important, may receive less attention. It was suggested that the creation of a national repository that gathers evidence and disseminates it across the researcher and policy communities would help to improve KT. A health researcher stated:

*There is need to put up systems and strategies for creating national health knowledge hubs with appropriate standard operating procedures for access and storage and must be accessible and available to researchers and policy-makers. Health research results are there not to be packed, but to help us create evidence and inform policies, so for that reason we are not able to do that, and so this has remained part of the complex challenge of translating evidence to policy.* [KI #2, Health Researcher]

In addition, some health researchers noted that KT was limited because there are not adequate platforms through which both health researchers and policy-makers can communicate on innovative ways to incorporate research findings during the different phases of policy formulation. Some health researchers indicated a need to strengthen platforms where research findings, in addition to producing the required reports, are regularly shared with the policy-makers. Direct engagement platforms such as the parliamentary committee on health, where researchers would present findings to lawmakers, were however said to be inadequate in the KT-to-policy process. A health researcher and a policy-maker, respectively, remarked:

*I think that knowledge translation into policy is extremely limited because there have not been platforms that are integrated and coordinated where this is done. But I think that there should be platforms where there is a policy engagement and others where researchers and policy-makers need to interact and engage each other.* [KI #2, Health Researcher]

*Quite often we are not part of stakeholder consultation, and yet we are expected to come and debate these laws and policies, and approve. That is why we have bad laws, because people do not have knowledge or an idea of what they are doing. So how do we expect people to make the law when they don’t understand the issues around it?* [KI #3, Policy-maker]

Another health researcher indicated:

*There is a gap in Zambia because knowledge translation is being embarked on now. I was saying that knowledge translation is important. Secondly, there seems to be a gap because there are a lot of research results that we have which I think have not been implemented into policy, and why is that? it is because for all these years we have not had a platform to use to ensure that there is knowledge translation.* [KI #10, Health Researcher]

## Discussion

The study findings show the complexity and insufficient cohesion of the current KT process in Zambia despite positive efforts over the years. The document review highlights the fact that the promotion of KT in most research governing policies through systems to harness local research is still inadequate. The document review further shows that there are efforts to improve the health research environment through regulation, coordination and institutional strengthening. However, the policies should go beyond prescribing a research environment, to focus more on specific aspects of KT such as evidence generation, synthesis, dissemination and implementation [18]. Furthermore, policies should seek to address the complex structure, iterative nature and inclusiveness of both the KT and policy processes [10, 19].

The Zambian government acknowledges the strategic role of KT in attaining national health goals, as evidenced by the creation of the NHRA by an act of parliament. The NHRA has made considerable progress in providing a platform for engagement of policy-makers, health researchers, the media and other interested stakeholders through annual research conferences. The value of creating space for dialogue between policy-makers and researchers to facilitate exchange during the policy-making process is highlighted by Nutley el al. [20]. Whilst the conferences by the NHRA are ideal for dissemination of research findings, there is a need for platforms that promote consistent engagement between policy-makers and researchers to allow for iterative and continuous exchange between the KT and policy processes. Suggestions to this effect may take the form of structured committees or working groups. An open regular update and exchange forum including the media and civil society would ensure diverse input, building trust and shared insight to ensure relevance for population health outcomes [21, 22].

Prior to the NHRA, early efforts to govern national-level KT in Zambia culminated in the creation of the Zambia Forum for Health Research (ZAMFOHR). This platform was created with the help of the WHO Evidence-Informed Policy Network (EVIPNet). Lessons from setting up the ZAMFOHR platform, still valid today, highlight the value of strategic leadership to manage linkages between policy-makers and researchers, building capacities in knowledge synthesis, communication and brokering [12]. Lessons from ZAMFOHR underscore the importance of developing coherent national/institutional KT strategies that are embedded within KT structures across various governance levels to feed into policy development and decision-making processes [23, 24].

KT efforts in Zambia have lagged due to limited investment in the national health research systems (NHRS). According to Kapata et al., this can frustrate health systems in their quest to achieve desired population health goals, because the NHRS may not adequately generate the required evidence to inform policy [25]. Furthermore, the lack of integrated and coordinated knowledge hubs hinders KT and development of the NHRS. Various researchers and routine data collection systems are gathering huge amounts of data on similar public health domains but are underutilized and disconnected on otherwise common aspects [26]. To improve KT, the safeguarding of knowledge generated by various health researchers and routine data systems must be ensured through the creation and linking of common data repositories and systems in order to coordinate knowledge creation, translation and use among various stakeholders.

In terms of research priorities, the need for new data must be appropriately balanced with the use and quality improvement of existing data. Researchers and policy-makers each have their own dependencies, which may amount to vested interests. Such are legitimate if open for discussion. Donors influence research through their conditions for funding, which may be more donor country- or global programme-based and thus a concern for country relevance [27, 28]. This includes donor programme-associated commercial consultancies, which may be largely uncritical towards methodologically weak terms of reference and are not held responsible for the changes that are effected after their departure. Here it would be better for donors to assign relevant in-country university and independent research institutions [29]. In addition, the government should consistently commit funds to health research and strengthening of the NHRA’s capacity to enhance the uptake of health research knowledge in policy-making through establishment of appropriate regulations and incentives.

KT occurs in a complex social system of interactions among various stakeholders. Possible interventions to enhance this process should be collaborative and two-way in nature, involving both the policy-makers and researchers. Strategies to improve evidence use in public health policy-making include regular contact with knowledge brokers to increase access to evidence, developing skills in appraisal and integration of evidence, and strengthening networks and exploring organizational factors to build organizational cultures receptive to embedding evidence in practice [30]. Knowledge translators need to identify key messages for different target audiences and to fashion these in language and KT products that are easily assimilated by different audiences [31]. A number of reviews provide a wide range of options for bridging the gap between policy/decision-makers and researchers [24, 30]. These reviews suggest that efforts to undertake translation of knowledge into policy are likely to produce desired results if they adopt KT strategies informed by an assessment of the local context by identifying potential enablers and how to overcome barriers.

When it comes to evidence/knowledge use in policy-making, it is also important to consider the models of policy-making that are being used. The different types of research evidence and their characteristics should equally be considered. Different forms of research evidence may serve best for certain kinds of policy decisions and policy-makers [32]. However, in much of the prevailing literature, the term health research evidence is very broad and could be subdivided into, for example, clinical, implementation and systems research. For knowledge, one could make a distinction between health outcome and managerial knowledge. For policy, however, this may be aligned with specific current policy and strategic recommendations or with adding insight to improve the quality of routine information systems. This could be anything from promoting a new and strengthened approach to a disease or condition or a specific problem, to a more general input to broader concerns for overall population health. In all contexts, general KT principles are applicable if the intention is widespread adoption of research evidence in the policy and decision processes.

## Study strength and limitations

This study was a qualitative study which allowed for detailed collection of data regarding the process of health research KT into policy in Zambia. However, though the study facilitated a rich description of the KT process, the responses given by participants could not be measured. Furthermore, the data collection from the policy-makers was a challenge due to their busy schedules. For this reason, we could not interview as many as we would have wanted. However, our having access to parliamentarians who sit on the parliamentary health committee compensated for our inability to interview a larger number of policy-makers. Through the diverse yet fairly small group of key informants, a number of important conditions for an effective KT process were identified. The study provides a rich perspective of some of these important conditions as well as challenges facing KT in Zambia.

## Conclusion

KT can address many of the challenges faced in the health sector in Zambia by implementing known policy interventions informed by available health research evidence/knowledge. Health research KT into policy remains a challenge in Zambia despite positive efforts over the years, owing to a number of contextual factors that shape the uptake of health research. These include, among others, limited resources, lack of space for dialogue, continued policy–researcher divide and lack of coordinated national knowledge hubs. There is a need for national health knowledge hubs with appropriate standard operating procedures that should be accessible to both researchers and policy-makers. Furthermore, there is a need to create more spaces for dialogue between health researchers and policy-makers as well as continuous strengthening and monitoring of KT capacity. Efforts that strengthen coordination of the process of health research KT among health researchers, policy-makers and key stakeholders will be vital in obtaining the full benefits of local evidence in improving the health of Zambians.

## Data Availability

The field notes and interview recorded verbatim and transcripts generated during data collection and analysis of this study are not publicly available to ensure the confidentiality and anonymity of the participating organizations and participants.

## Abbreviations

CIDRZ: Centre for Infectious Disease Research in Zambia
KT: Knowledge translation
KTA: Knowledge to action
MoH: Ministry of Health
NHRA: National Health Research Authority
TWG: Technical working group
WHO: World Health Organization
ZNPHI: Zambia National Public Health Institute
